# Linking pre-existing biorepositories for medical research: the PopGen 2.0 Network

**DOI:** 10.1007/s12687-019-00417-8

**Published:** 2019-03-29

**Authors:** Wolfgang Lieb, Gunnar Jacobs, Andreas Wolf, Gesine Richter, Karoline I. Gaede, Jeanette Schwarz, Norbert Arnold, Ruwen Böhm, Alena Buyx, Ingolf Cascorbi, Andre Franke, Christine Glinicke, Janka Held-Feindt, Ralf Junker, Holger Kalthoff, Hans-Heiner Kramer, Frank Leypoldt, Nicolai Maass, Walter Maetzler, Sandra May, H. Maximilian Mehdorn, Christoph Röcken, Clemens Schafmayer, Martin Schrappe, Stefan Schreiber, Susanne Sebens, Ulrich Stephani, Michael Synowitz, Jörg Weimer, Peter Zabel, Ute Nöthlings, Christian Röder, Michael Krawczak

**Affiliations:** 1 Institute of Epidemiology Kiel University and PopGen Biobank, University Hospital Schleswig-Holstein UKSH, Campus Kiel Hs. 1, Niemannsweg 11, 24105 Kiel, Germany; 2 Institute of Medical Informatics and Statistics, Kiel University, Kiel, Germany; 3 Division of Biomedical Ethics, Institute of Experimental Medicine, Kiel University, Kiel, Germany; 4 BioMaterialBank Nord, Department of Medicine, Leibniz Lung Center for Medicine and Biosciences, Borstel, Germany; 5 Institute of Clinical Chemistry, Kiel University, Kiel, Germany; 6 Institute of Clinical Molecular Biology, Kiel University, Kiel, Germany; 7 Department of Gynecology and Obstetrics, University Hospital Schleswig-Holstein Campus Kiel, Kiel, Germany; 8 Institute of Experimental and Clinical Pharmacology, Kiel University, Kiel, Germany; 9 Ethics Committee of the Medical Faculty, University of Kiel, Kiel, Germany; 10 Department of Neurosurgery, University Hospital Schleswig-Holstein Campus Kiel, Kiel, Germany; 11 Institute for Experimental Cancer Research, Kiel University, Kiel, Germany; 12 Department for Congenital Heart Disease and Pediatric Cardiology, University Hospital Schleswig-Holstein Campus Kiel, Kiel, Germany; 13 Department of Neurology, University Hospital Schleswig-Holstein Campus Kiel, Kiel, Germany; 14 Institute of Pathology, Kiel University, Kiel, Germany; 15 Department of General and Thoracic Surgery, University Hospital Schleswig-Holstein Campus Kiel, Kiel, Germany; 16 Department of Pediatrics, University Hospital Schleswig-Holstein UKSH, Campus Kiel, Kiel, Germany; 17 Department of Internal Medicine I, University Hospital Schleswig-Holstein Campus Kiel, Kiel, Germany; 18 Department of Neuropediatrics, University Hospital Schleswig-Holstein Campus Kiel, Kiel, Germany; 19 Department of Pneumology, Leibniz Lung Center for Medicine and Biosciences, Borstel, Germany; 20 Department of Nutrition and Food Sciences, University of Bonn, Bonn, Germany

**Keywords:** Biobank, Governance, Ethics, Data management, Quality assurance

## Abstract

The significance of human biorepositories for modern medical research, particularly for comprehensive population-based genetic analyses, is constantly growing. While large and centralized institutions are usually considered best suited to meet the increasing demand for high-quality “biobanks,” most medical research institutions still host rather heterogeneous and fragmented biobanking activities, undertaken by clinical departments with oftentimes rather different scientific scope. Undoubtedly, most clinicians and medical researchers would appreciate infrastructural support in terms of the storage and handling of their biosamples, but they are also likely to expect access to their samples avoiding extensive formal requirements. We report on the establishment of the PopGen 2.0 Network (P2N), an overarching alliance of initially seven biobanks from Northern Germany which adopted a joint but lean governance structure and use-and-access policy for their samples and data. In addition, the members of P2N have pursued an intense collaboration on ethical, legal and social issues and maintain a common IT infrastructure. The implementation of P2N has substantially improved the prospects of biobank-based research at the participating institutions. The network may thus serve as a role model for similar initiatives geared at linking pre-existing biorepositories for the benefit of research quality, efficiency, and transparency.

## Background

Biorepositories are an indispensable prerequisite of modern biomedical, particularly omics-based, research. For example, the genetic characterization of large clinical and population-based cohorts has yielded an ever improving overview of the genetic basis of many human diseases. These analyses would not have been feasible without the standardized collection, storage and analysis of medical data and biological samples by dedicated and suitably equipped institutions. In addition, biobanks also bear great potential in the field of Community Genetics, as defined by Ten Kate et al. (2010). Thus, comprehensive population- and clinical care–based collections of biosamples not only form the basis of epidemiological studies of health and disease. They are also required to assess the efficiency of genetic screening strategies, to study the genetic “make-up” of diseases in particular patient groups, and to complement registers for genetically determined conditions. In consequence, the scientific value of biorepositories has been widely recognized, causing the number of such institutions to grow worldwide (Bernemann et al. 2016; Kinkorova 2015).

At the same time, the demands placed on biorepositories have increased as well in multiple ways, thereby stimulating the drawing-up of guidelines for biorepositories by several professional organizations, including ISBER (International Society for Biological and Environmental Repositories) (Campbell et al. 2012) and the US National Cancer Institute (National Cancer Institute 2016; Vaught et al. 2011). The most important issues addressed by these guidelines are the proper documentation of the collection, the pre-analytical handling and the storage conditions of the samples (National Cancer Institute 2016), the governance structure and the use-and-access policy, and the IT and ELSI (ethical, legal, social issues) frameworks (Campbell et al. 2012; Langhof et al. 2017; National Cancer Institute 2016).

While large centralized institutions are often deemed most suitable to meet the above requirements and to better foster international collaborations through their greater visibility, for example, in international biorepository catalogues, many medical research institutions still sustain heterogeneous and fragmented biobanking activities. Individual repositories often grew out of individual research interests, are therefore focused upon one or a few diseases, and consequently belong to different (clinical) departments with different technical and organizational infrastructure. Moreover, the research groups involved are often responsible for both the collection and scientific use of their samples. This status quo reflects a somewhat ambivalent albeit popular attitude towards biobanking: While clinicians would undoubtedly appreciate infrastructural support to ensure high-quality storage and handling of their samples, they also expect access to their samples to proceed exclusively through them, with very limited to no formal requirements. Instead of setting up solitary infrastructures, it may thus be more sensible in many instances to develop and promote networks of pre-existing biobanks as a way of balancing local autonomy with the requirements of modern medical research. Furthermore, many aspects of biobanking, including IT and ELSI, can be handled more effectively by way of collaboration than through individual activities.

Taking the above considerations into account, the PopGen 2.0 Network (P2N) was established in 2011 as an organizational umbrella for seven pre-existing biorepositories in the most northern part of Germany, integrating various sample collections from the Kiel Campus of the University Hospital Schleswig-Holstein (UKSH) and the Leibniz Lung Centre for Medicine and Biosciences at Borstel, located 80 km south of Kiel.

## Foundations of P2N

In May 2010, the German Ministry of Education and Research (BMBF) launched a funding scheme entitled “centralized biomaterial banks (cBMB),” which was specifically geared at the networking of pre-existing biomaterial collections from patient-based medical research. The call was intended to help German biobanks to overcome possible shortcomings in terms of sample handling and data management, with a particular view to fostering German contributions to the EU-funded Biobanking and BioMolecular Resources Research Infrastructure (BBMRI).

Grouped around the local PopGen biobank (Krawczak et al. 2006), which holds approximately 160,000 samples from > 80,000 individuals, seven biorepositories located at the UKSH Campus Kiel and the Research Center Borstel applied for funding under the BMBF call to form the P2N. In addition to PopGen, the participating institutions included the Kiel Comprehensive Cancer Center North together with the Institute for Experimental Cancer Research; the Department of Neurosurgery; the Institute of Pathology; the Institute of Experimental and Clinical Pharmacology; a consortium comprising the Departments of Neuropediatrics, Pediatrics, Human Genetics, and Gynecology and Obstetrics; as well as the Research Center Borstel.

Prior to the initiation of P2N, the respective biobanks had rather different data management systems, IT infrastructures and storage facilities. Consent documents, quality management and access policies also varied widely. The central goal of P2N was to harmonize these disparate procedures and practices by way of establishing a central governance structure. The network was intended not only to ensure high-quality biobanking but also to actively promote the use of the samples and data of the member collections for scientific research. Together with four other sites in Germany (Aachen, Berlin, Heidelberg, and Würzburg), P2N successfully acquired funding from the BMBF call in July 2011. The implementation of professionalized biobanks at the five sites provided the foundation for a German Biobank Node (GBN), which now forms the national bridgehead for activities within the BBMRI–European Research Infrastructure Consortium (http://www.bbmri-eric.eu). In its capacity as BBMRI-ERIC hub, GBN aims at the nationwide harmonization of probe management, quality control, and IT as building blocks of a coordinated exchange of biosamples and data with other biobanks or with external partners.

The achievements made by P2N during the 5-year cBMB funding period (2011–2016) are summarized in the following sections.

## Governance structure

The seven founder biobanks of P2N established a joint governance structure and agreed to abide to the respective stipulations by way of a formal collaborative agreement. Noteworthy, three additional biobanks have since joined the network.

**Fig. 1:**
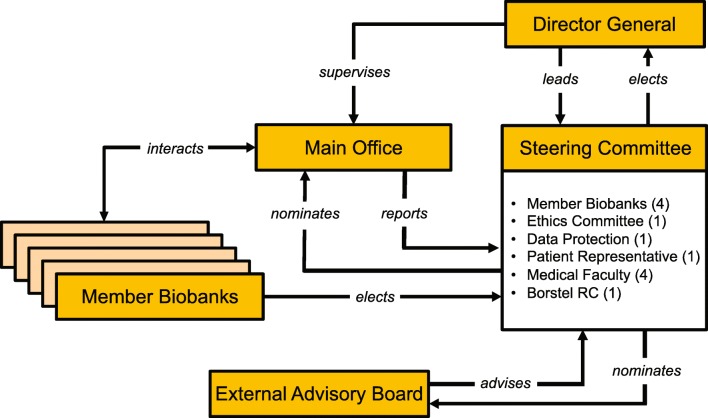
Governance structure of P2N; RC Research Center. The number of members of the Steering Committee delegated by each stakeholder group is given in brackets

The different components of the P2N governance structure and their interrelationships are depicted in Fig. 1. All routine affairs of the network are dealt with by the *Main Office***,** which also handles the requests made for data and samples of the *Member Biobanks*. The Main Office also coordinates the quality management activities of the network, administers its website (https://www.uksh.de/p2n/), and coordinates the public involvement of P2N.

The network is chaired by a *Director General* who supervises the Main Office and represents P2N both internally and externally. The Director General is elected by the *Steering Committee* who advises them on running the network and ultimately has to approve all requests for samples and data (for details, see below). The Steering Committee comprises representatives of the Member Biobanks, the local ethics committee, the UKSH data protection office, a patient organization, the Medical Faculty of Kiel University, and the Research Center Borstel.

## Use-and-access policy

**Fig. 2:**
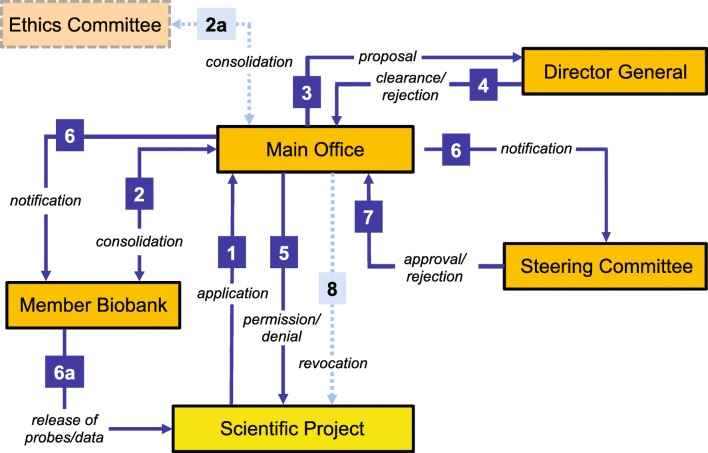
P2N use-and-access process; numbers referring to different stages of the use-and-access process are explained in detail in the text

The P2N Member Biobanks agreed upon a joint use-and-access policy (Fig. 2) and implemented a corresponding application process, using a single form for all data and sample requests. This form is issued, received and processed by the Main Office (step 1). All requests are forwarded simultaneously to the affected P2N Member Biobank(s) for scientific evaluation (step 2) and to the local ethics committee (step 2a). Both institutions also assess whether the intended research is in accordance with the informed consent given by the sample donors or data subjects. As a matter of principle, the Member Biobanks have the final say about the use of their samples and data. Furthermore, every request must be backed by a positive vote from the responsible ethics committee. To ensure timely evaluation and decision making, both of which are critical for the acceptance of a network like P2N, the Director General may choose between two types of decision process, comprising either a regular or an extended path.

Along the regular path, the Director General makes a preliminary decision about the request (steps 3 and 4) given that there is clearance by the affected Member Biobank(s) and the local ethics committee. This preliminary decision is communicated to the applicant (step 5) and circulated among the Steering Committee members for approval (steps 6 and 7). In the expectedly rare instance that the Steering Committee overrules the decision by the Director General, the preliminary approval is revoked (step 8). Along the extended path, the primary decision about a request is made by the Steering Committee directly.

The regular path was implemented because it provides a satisfactory balance between the functionality, and hence acceptance, of the P2N governance on the one side, and the interests of critical stakeholders on the other. Based upon our own experience from dealing with > 100 requests per year, more than 95% of cases are so straightforward that the regular path legitimately can be chosen. Moreover, since requests handled along the regular path are usually clear-cut, we expect contradictory verdicts by the Director General and the Steering Committee to be extremely rare.

## Quality management of the network

Using the roXtra QM software package (Rossmanith, Göppingen, Germany) for document control and management, a computerized quality management system (QMS) was set up by P2N. Standard operating procedures (SOP) and data protection manuals released by the individual P2N partner biobanks are also shared, using the same document management system (DMS). The roXtra DMS allows centralized web-based documentation and version control. It guarantees access at any time to the most recent document versions, provides an email service to invite users to retrieve new documents and electronically documents reading receipts. The DMS workflow includes centralized recording of the document history comprising all stages of compilation, reviewing, and approval of documents as well as the withdrawal and archiving of outdated versions. Task management in roXtra proceeds through the campus-wide intranet of UKSH to which external partner biobanks are connected via VPN. Software support, training, and user administration come from the institutional IT department.

The P2N QMS manual was structured according to, and complies with, DIN/ISO 9001. In November 2015, the P2N QMS was adapted to the new DIN/ISO 9001:2015. Since P2N is a network of pre-existing biobanks, its QMS is focused upon overarching issues such as governance, ELSI and administration, including the use-and-access procedures. It does not regulate any biobank-specific procedures because these mostly had been dealt with before the formation of P2N. Nevertheless, all biobank-specific documents are also catalogued centrally in the DMS. Notably, some P2N Member Biobanks are bound by quality stipulations made, for example, by other biobanking consortia with own rules and SOPs.

**Fig. 3:**
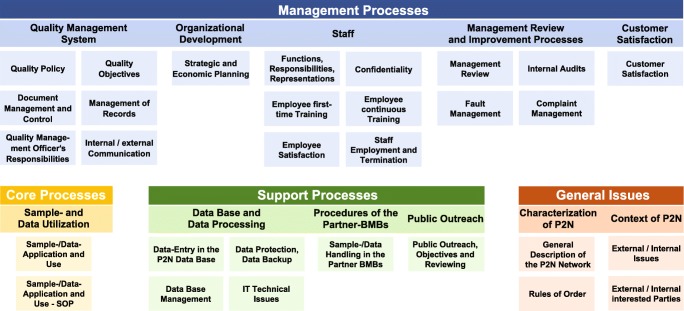
Quality management system of P2N; the different color schemes signify different process classes. The core process class of P2N is confined to sample and data application and sharing only because the responsibility for sample and data collection, handling and storage rests with the individual P2N Member Biobanks. SOP standard operating procedure, IT information technology, P2N PopGen 2.0 Network

The structure of the P2N QMS manual can be summarized by a process map containing a schedule of responsibilities (Fig. 3). The QMS mainly comprises

1. *general network documents* on the scope, governance, and operational environment of P2N
2. *management processes* addressing quality issues and communication, strategic and personnel planning, opportunities and risk management, performance evaluation, and client satisfaction
3. *core processes* regulating the operationalization of sample and data application and release
4. *support processes* addressing technical issues, data protection, sample and data handling and quality assurance by Member Biobanks, network public outreach, and requirements

Together with other biobanks in Germany, P2N has actively participated in a quality management initiative launched by the GBN and the TMF. The initiative was also backed by the German Biobank Alliance (GBA), a successor to the cBMB program that has been funded by the BMBF since 2017. The GBA is currently developing quality measures and generic SOPs for both liquid and tissue biomaterial handling. They are also involved in defining the relevant aspects of a new biobanking-specific DIN/ISO norm and contribute to the development of the IT infrastructures required for sustaining the national and international exchange of biosamples and data.

## Biobank information management system CentraXX

The CentraXX software (Kairos GmbH, Bochum, Germany) furnishes P2N Member Biobanks with a versatile Biobank Information Management System (BIMS). The CentraXX client-server architecture provides a knowledge portal allowing donor-centered views on all available sample and donor information. Personal data identifying individual donors are pseudonymized by an external trusted third party (Section Epidemiology of Health Care and Community Health, Institute for Community Medicine, University Medicine Greifswald, Germany) connected to the BIMS so that all other data are essentially stored in anonymous form. Initially tailored to the administration of sample data (e.g., type, amount, concentration, chain of custody) and infrastructure data (e.g., sample containers, location element, stock locations), the functionality of CentraXX has since been extended to the documentation of additional information on samples (e.g., freeze–thaws, measurement profiles) and donors (e.g., ICD, OPS, electronic case report forms). In addition, system interfaces allow the enrichment of donor records with treatment data from the UKSH hospital information system, provided that the donor has given appropriate consent. P2N has also agreed upon a minimal dataset for all samples stored by the Member Biobanks (Table 1). Missing values are allowed to ensure comprehensive data intake, particularly from external sources. In conclusion, use of a joint BIMS allows the identification of sample donors and the integration of their data across P2N Member Biobanks.

**Table 1:** Minimal data set of the PopGen 2.0 Network (P2N)

Sample-related data	
Identifier of host biobank	
Unique P2N sample identifier	
Host biobank-specific sample identifier(s)	
Kind of sample	
Date of sampling	
Date of sample processing	
Initial amount of material	
Remaining amount of material	
Storage location	
SPREC 2.0 (Lehmann et al. 2012) for fluid samples	
Type of sample	
Type of primary container	
Pre-centrifugation	
Centrifugation	
Second centrifugation	
Post-centrifugation delay	
Long-term storage	
SPREC 2.0 (Lehmann et al. 2012) for solid samples	
Type of sample	
Type of collection	
Warm ischemia time	
Cold ischemia time	
Fixation/stabilization type	
Fixation time	
Long-term storage	
Donor-related data	
Unique donor identifier (i.e., the pseudonymized master person index)	
Host biobank-specific donor identifier(s)	
Donor year of birth	
Donor sex	
ICD code of main donor diagnosis at time of sampling	
Associated MeSH terms	
Associated MIM codes	

All attributes are mandatory but missing values are allowed in order to facilitate data entry from third party sources

*SPREC* Standard PRE-analytical Code, *ICD* International Classification of Disease, *MeSH* Medical Subject Headings, *MIM* Mendelian Inheritance in Man

## ELSI

Biobanks are facing a number of important ethical issues, including the necessity to develop appropriate informed consent documents, the handling of incidental findings as well as ownership issues, and the sharing of samples. The ELSI activities of P2N started out from an inventory of the consent documents used by the Member Biobanks. Subsequently, two new forms were developed for prospective use in P2N, namely a broad consent form covering the research use of medical data and leftover biomaterial from clinical routine at the UKSH Campus Kiel and a modular template for study-specific sample collections with defined research questions. Key features of both consent forms are provided in Table 2 and are detailed below.

**Table 2:** General and specific key features of two informed consent forms developed within P2N

General features	
Unlimited storage of data and biomaterial	
Transfer of property rights to local university hospital (UKSH)	
Non-reporting of incidental findings	
Right to withdraw	
Type of scientific analyses permitted (i.e., genetic, biochemical, tissue-based)	
Permission to transfer data and samples to third parties (e.g., collaborators or research databases)	
Permission to re-contact donors	
Permission to contact treating physicians	
Features of the modular, study-specific consent	Features of the broad consent
Definition of specific research purpose	Explanation of broad consent
Scope of consent (i.e., defined disease, disease groups, or biomedical research in general)	Non-reporting policy
Type and amount of biomaterial	Coverage of leftover samples from clinical routine

### Modular, study-specific consent

Since many parts of the informed consent documents required by P2N Member Biobanks were likely to be similar, we developed a modular consent form to help researchers to create such documents more easily and to promote more efficient evaluation of the documents by research ethics committees. In order to meet the specific requirements of individual Member Biobanks while taking the similarities between potential research projects into account, the modular informed consent form combines optional and mandatory elements (Table 2). More specifically, mandatory parts as agreed upon by the network reflect general P2N policies regarding the transfer of property rights, the withdrawal process, permission to re-contact donors, permission to contact treating physicians, duration of storage and research, handling of incidental findings, types of analysis permitted (i.e., genetic, biochemical, tissue-based), and the permission to transfer data and samples to third parties, particularly international databases. Optional parts of the template were designed so as to allow for the specificities of individual projects, including the definition of the specific research purpose, its scope (single disease, disease groups, or biomedical research in general), and the type of biomaterial involved.

### Broad consent

In addition to supporting the project-specific collection of biomaterial, P2N also spearheaded the local implementation of healthcare-embedded biobanking, defined as the large-scale collection and storage of leftover samples from clinical routine. Since most scientific questions, which can potentially be addressed by the analysis of such biomaterial are unknown at the time of collection, seeking broad consent from patients for this kind of activities appeared most sensible and, if done conscientiously, ethically acceptable.

In order to inform patients adequately about healthcare-embedded biobanking, we developed a short information brochure. The intelligibility of the information and the general acceptance of the broad consent were evaluated in a pilot study carried out at the Comprehensive Centre of Inflammation Medicine at UKSH Campus Kiel (Richter et al. 2018). In essence, patients generally accepted the broad consent policy and agreed to both an unlimited storage and a wide use of their samples and data for medical research. While our study showed that participants who consented had both modest subjective and objective understanding of the information brochure provided, a large majority had strong prosocial reasons to donate (Richter et al. 2018).

Relevant staff was trained for the campus-wide implementation of the broad consent by an information campaign introducing them to the specificities of the content and handling of the consent form. As an additional trust-building measure, P2N provides a telephone hotline, manned with a trained ethicist, to answer patient questions about the broad consent.

Requests for withdrawal from P2N Partner Biobanks are processed by the affected biobanks themselves because, in most cases, such requests are sent directly to them. If a request is sent to the P2N Main Office, it is forwarded to the responsible Member Biobank immediately.

### Campus-wide ELSI service

Finally, P2N also set up a campus-wide ELSI service at Kiel covering all ethical domains of biomedical research. For example, the service helps researchers preparing research proposals as well as drafting information and consent material. The service also offers general evaluation of research proposals with regard to ethical acceptability. Implementation of the ELSI service has already led to a notable reduction of the processing time of such proposals by the local ethics committee.

## Concluding remarks

The significance of biobanks for both medical research and clinical care is constantly increasing, a development that is paralleled by the emergence of ever more stringent requirements and regulations for such institutions. With funding from a 5-year grant by the German Federal Ministry of Education and Research, we successfully established the PopGen 2.0 Network (P2N), an organizational umbrella for seven pre-existing biobanks at University Hospital Schleswig-Holstein (UKSH) Campus Kiel and the Leibniz Lung Centre for Medicine and Biosciences, Borstel. P2N and its expertise in biobanking have gained wide-spread recognition by the Medical Faculty and the UKSH Campus Kiel; the network has since grown to comprise ten Member Biobanks. In addition to its local acceptance, P2N has since also received and processed several hundred samples and data requests from national and international research groups. In close collaboration with its Member Biobanks, P2N implemented a comprehensive governance structure, including a network-wide use-and-access policy, alongside a common IT infrastructure and an overarching quality management system. Together with the sustainable ELSI support provided, the developments within P2N greatly improved the prospects of biobank-based research at the participating institutions and beyond. The joint successful infrastructural development at UKSH Campus Kiel and the Research Center Borstel led to a long-term cooperative agreement between the two institutions and attracted sustainable long-term funding of P2N (as a core facility on campus) by the Medical Faculty of Kiel University in 2017. In view of its successful development, P2N may serve as a role model for similar initiatives geared at linking pre-existing biomaterial collections for the benefit of research quality, efficiency, and transparency.
